# Changes in Serum Levels of Matrix Metalloproteinase-1 and Tissue Inhibitor of Metalloproteinases-1 in Patients with Essential Hypertension

**DOI:** 10.3390/bioengineering9030119

**Published:** 2022-03-15

**Authors:** Krasimir Kostov, Alexander Blazhev

**Affiliations:** 1 Department of Pathophysiology, Medical University-Pleven, 5800 Pleven, Bulgaria; 2 Department of Biology, Medical University-Pleven, 5800 Pleven, Bulgaria; yalishanda9@gmail.com

**Keywords:** essential hypertension, matrix metalloproteinase-1, tissue inhibitor of metalloproteinases-1

## Abstract

Hypertension (HTN) is a leading risk factor for cardiovascular (CV) disease. Matrix metalloproteinases (MMPs) and their tissue inhibitors (TIMPs) are thought to be actively involved in the remodeling of the CV extracellular matrix (ECM) during hypertensive damage. Therefore, in this study, we aimed to investigate serum levels of MMP-1 and TIMP-1 in patients with essential HTN and compare them with those of normotensive individuals. We measured serum concentrations of MMP-1 and TIMP-1 in 60 patients with HTN and 20 healthy controls using an ELISA. The obtained results showed that in patients with HTN, the mean levels of MMP-1 (1.82 ± 0.9 ng/mL) were significantly higher (*p* = 0.03) than the mean levels in the control group (1.19 ± 0.7 ng/mL). The levels of TIMP-1 in patients with essential HTN (0.44 ± 0.1 ng/mL) were also significantly higher (*p* = 0.005) than those in the control group (0.33 ± 0.1 ng/mL). In HTN, elevated serum MMP-1 levels may be associated with increased collagen degradation in the CV ECM, whereas elevated TIMP-1 levels may favor its accumulation and the development of pathological remodeling and fibrosis of the heart and arterial vessels.

## 1. Introduction

Hypertension (HTN) is one of the most prevalent diseases worldwide and is among the most important risk factors for cardiovascular (CV) and cerebrovascular complications [1,2]. The search for suitable biomarkers for CV remodeling and fibrosis may identify individuals at increased risk of developing hypertensive CV disease [3,4,5]. High blood pressure (BP) initiates changes in the extracellular matrix (ECM) of blood vessels [6] and the heart [7], leading to progressive vascular damage and heart dysfunction. In this regard, the monitoring of circulating levels of matrix metalloproteinases (MMPs) and their tissue inhibitors (TIMPs) can provide important information about hypertensive CV remodeling, which take place at the tissue level [8,9].

MMPs are a family of zinc-dependent endopeptidases that regulate a variety of cellular functions [10]. They are produced by multiple tissues and cells (fibroblasts, endothelial cells, vascular smooth muscle cells, macrophages, neutrophils, and lymphocytes) [8]. MMPs are classified according to their substrate specificity in the following: collagenases (MMP-1, MMP-8, MMP-13, and MMP-18); gelatinases (MMP-2 and MMP-9); stromelysins (MMP-3, MMP-10, and MMP-11); matrilysins (MMP-7 and MMP-26). In addition, membrane-type MMPs (MT-MMPs), including transmembrane (MMP-14, MMP-15, MMP-16, and MMP-24) and membrane anchored (MMP-17 and MMP-25) have been defined [11]. The activity of MMPs is regulated at multiple levels, including mRNA expression and activation of the proenzyme to the active form, but mainly by inhibitory actions of four TIMPs (TIMP-1, TIMP-2, TIMP-3, and TIMP-4) [12].

The heart and blood vessels contain MMPs with varying degrees of specificity that can degrade different components of the ECM. The main MMPs present in blood vessels are collagenases (MMP-1 and MMP-13), which degrade collagen (COL) types I, II, and III, gelatinases A (MMP-2) and B (MMP-9), which degrade denatured COL (gelatin) and COLs type IV and V, and stromelysins (e.g., MMP-3), which degrade adhesive molecules such as laminin, fibronectin, nonfibrillar COLs, and proteoglycans [13]. MMPs that have been identified in the heart are MMP-1, MMP-3, MMP-8, MMP-13, MMP-2, MMP-9, MMP-12, MMP-28, and MT1-MMP/MMP-14 [14].

In the last years, MMP-1 and TIMP-1 (the inhibitor of soluble MMPs but not of MT-MMP), have been one of the most studied for hypertension-mediated damage to vessels and target organs [8]. MMP-1 cleaves both ECM and nonECM substrates such as COL, gelatin, laminin, complement component C1q, interleukin-1β, and tumor necrosis factor-α, suggesting a crucial role in inflammatory and fibrotic responses [15,16]. In addition, the changes in TIMP-1 have been proposed as a mediator of CV remodeling and fibrosis [17]. These findings suggest that monitoring MMP-1 and TIMP-1 levels may provide important information about the ongoing adverse processes of arterial and left ventricular remodeling and fibrosis in patients with HTN [9,17,18,19,20].

Based on the above, in the present study, we aimed to compare serum levels of MMP-1 and TIMP-1 between patients with essential HTN and normotensive individuals.

## 2. Materials and Methods

### 2.1. Characteristics of the Study Population

The study population consisted of 20 healthy normotensive individuals (the control group) and 60 patients with essential HTN (the hypertensive group), who are on antihypertensive therapy and are periodically admitted for control and monitoring of the disease at the Dr. Georgi Stranski University Hospital in Pleven. The clinical characteristics of the groups are shown in Table 1.

### 2.2. Blood Pressure Measurement

BP was measured by the classical method, on the left arm in a sitting position, after 5–10 min rest. The assessment of the arterial HTN was made according to the 2018 ESC/ESH Clinical Practice Guidelines for the management of arterial HTN [21]. Normal BP was defined as SBP 120–129 mmHg and DBP 80–84 mmHg. HTN was defined as SBP ≥ 140 mmHg or DBP ≥ 90 mmHg.

### 2.3. Immunological Assays

To measure serum levels of MMP-1 and TIMP-1, blood was drawn into serum tubes and centrifuged at 2500 rpm for 10 min. The concentrations were measured by the ELISA method using commercially available kits from R&D Systems, Minneapolis, MN, USA (MMP-1, cat no. DMP100 and TIMP-1, cat no. DTM100). Serum samples were assayed at 450 nm on a Coulter Microplate Reader UV Max (Molecular Devices Corp., Menlo Park, CA, USA).

### 2.4. Statistical Analysis

The analysis of the statistical data was performed by using SPSS 23.0 software (SPSS, Inc., Chicago, IL, USA). The differences between the groups were assessed by an unpaired Student’s t-test. The data were expressed as mean ± SD. *p* values < 0.05 were considered statistically significant.

## 3. Results

### 3.1. Comparison of Serum Levels of MMP-1 between the Hypertensive Group and the Control Group

**Figure 1:**
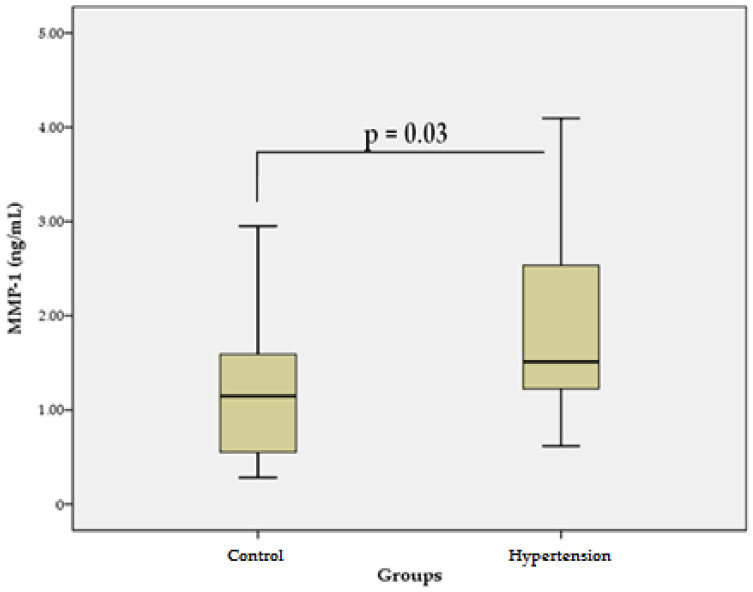
Serum levels of MMP-1 in the hypertensive group vs. control group. Data are represented as mean ± SD.

The results of our study in patients with essential HTN revealed that the mean serum levels of MMP-1 (1.82 ± 0.9 ng/mL) were significantly higher (*p* = 0.03) than the mean levels in the control group (1.19 ± 0.7 ng/mL) (Figure 1). These data suggest that MMP-1 could be associated with the increased degradation of COL and pathological CV remodeling in primary HTN.

### 3.2. Comparison of Serum Levels of TIMP-1 between the Hypertensive Group and the Control Group

**Figure 2:**
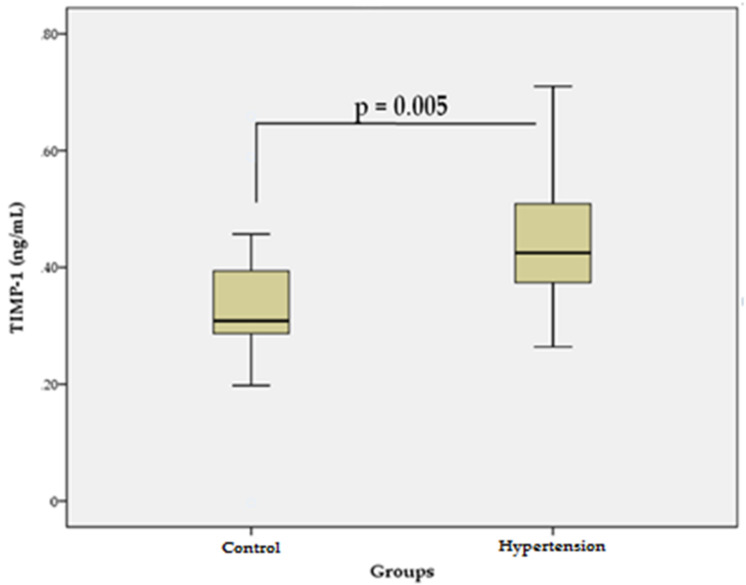
Serum levels of TIMP-1 in the hypertensive group vs. control group. Data are represented as mean ± SD.

The levels of TIMP-1 in patients with essential HTN (0.44 ± 0.1 ng/mL) were significantly higher (*p* = 0.005) than those in the control group (0.33 ± 0.1 ng/mL) (Figure 2). These data suggest that TIMP-1, which inhibits the activity of many MMPs, may favor excessive accumulation of COL in the CV ECM, and thus, may contribute to the development of vascular fibrosis and cardiac dysfunction in primary HTN.

## 4. Discussion

Because changes at the cellular level are reflected in body fluids, the determination of MMPs and TIMPs in the blood can be used as a noninvasive approach to diagnose and monitor structural changes in a number of diseases [22]. Changes in serum levels of MMPs and TIMPs reflect dysregulation of the synthesis and degradation of ECM proteins, which is a key process in the pathophysiology of vascular remodeling in HTN [23,24]. Hypertension-mediated vascular changes are associated with dynamic interactions between vasoactive substances, hemodynamic stimuli, and the vascular ECM [6,25]. All of these interactions initiate changes in MMP/TIMP activity in the vascular wall, which alters the balance between synthesis and degradation of ECM components [26].

The effects of MMPs on vascular fibrosis in HTN may vary, with both inhibitory and stimulatory modulation observed. This is probably related to the activation of various MMP isoforms and signaling pathways. For example, overexpression of MMP-1 attenuates the processes of fibrosis, whereas activation of MMP-9 potentiates them [27]. In addition, MMP-1 can activate MMP-2 and MMP-9, initiating an activation cascade [28]. Our results suggest that higher levels of MMP-1 in patients with HTN (Figure 1) may reflect increased degradation of COL in the arterial wall. On the other hand, higher levels of MMP-1 may be associated with COL accumulation in the wall of the arteries due to activation of MMP-2/MMP-9 [28] and initiation of a profibrotic response [27]. These findings are supported by our previous studies in hypertensive patients, in which MMP-2 and MMP-9 showed significantly higher serum levels [29]. MMP-2/MMP-9 activation through transforming growth factor-β/Smad (TGF-β1/Smad) signaling induces activation of myofibroblasts and increased leukocyte infiltration, leading to inflammation and vascular wall injury. Activation of MMP-2 leads to enhanced TGF-β1 signaling, increased production of COL I, II, and III by vascular smooth muscle cells and increased secretion of fibronectin, processes that lead to the accumulation of COL in the vascular wall. In addition, MMP-2 and MMP-9 enhance the release of TGF-β1, which in turn, stimulates TIMPs [27]. Our results support these findings because the serum levels of TIMP-1 in patients with essential HTN were significantly higher than those in the control group (Figure 2). Higher levels of TIMP-1 are probably associated with the compensatory inhibition of ECM degradation with COL accumulation and vascular fibrosis, which is due to the fact that TIMP-1 is a strong inhibitor of many MMPs [30].

High BP also leads to adaptive remodeling in the heart, which is characterized by intrinsic cardiomyocyte reorganization, and changes of ECM with increased COL deposition and interstitial fibrosis [8]. In patients with HTN, changes in ECM contribute to the development of structural and functional abnormalities that cause progressive cardiac dysfunction [31]. In them, normal levels of MMPs and TIMPs correlate with normal structure and function of the left ventricle (LV). Ahmed et al. showed that hypertensive patients who had normal LV structure and function had normal plasma levels of MMPs and TIMPs [24]. Patients with HTN with LV hypertrophy (LVH) had elevated levels of circulating TIMP-1 but decreased circulating levels of MMP-1 compared with patients without LVH [32]. Our data in patients with HTN show that they have both elevated levels of TIMP-1 and elevated levels of MMP-1. Lindsay et al. expressed the opinion that TIMP-1 levels are not elevated in HTN per se, but only in patients with diastolic dysfunction and fibrosis, and reported that elevated TIMP-1 levels are at odds with results showing an increased COL degradation [18]. This is not necessarily controversial, as more recent data show that TIMP-1 can trigger fibrosis independent of its MMP-inhibitory function. TIMP-1 can promote fibrosis of the myocardium by mediating the interaction between CD63 (cell surface receptor for TIMP-1) and integrin β1 on fibroblasts, activation and nuclear translocation of Smad2/3 and β-catenin, and the induction of de novo mRNA expression of COLs type I and III [17]. In addition, elevated levels of MMP-1 in our study support the findings of increased COL degradation in HTN. It is likely that initial changes in the MMP-1/TIMP-1 profile favor ECM accumulation and are associated with LVH and diastolic dysfunction. As the process progresses, increased degradation of ECM components in the LV may be a sign of transition to systolic myocardial dysfunction [33].

The strength of our study was that the determination of MMP-1 and TIMP-1 in the serum can be used as a noninvasive approach to diagnose and monitor structural changes in the CV system in HTN. A limitation of the study is that the average age in the control group differs from that in the hypertensive group. To some extent, this may lead to inaccuracies in the interpretation of the results, but it should be borne in mind that changes in MMP/TIMP levels depend mainly on the underlying CV pathology (e.g., HTN, heart attack, coronary heart disease, peripheral arterial disease, generalized atherosclerosis, stroke, heart failure, diabetes, etc.) and the degree of its manifestation, and not so much by age, sex, or race.

## 5. Conclusions

In summary, our results indicate that the mean serum levels of MMP-1 and TIMP-1 in patients with essential HTN were significantly higher than in the control group. These data suggest increased collagenolytic activity of MMP-1, which may be associated with adaptive changes in the CV ECM in response to sustained high BP. On the other hand, increased activity of MMP-1, as well as other MMPs in HTN, may be the cause of compensatory increases in TIMP-1 levels, which may subsequently lead to the development of CV fibrosis and LV diastolic dysfunction. Future studies of the relationship between MMP-1 and TIMP-1 levels with various parameters of CV morphology and function, as well as DNA sequence analysis of MMP-1 and TIMP-1, could provide more detailed information on the processes of CV remodeling and fibrosis in patients with primary HTN and give an additional insight into the population genetics and responsiveness to this significant disease.

## Figures and Tables

**Table 1:** Characteristics of the groups.

Examined Individuals (*n* = 80)	Control Group (*n* = 20)	Hypertensive Group (*n* = 60)
Sex, male/female	10/10	24/36
Age, years ^1^	47.9 ± 11.3	65.3 ± 11.5
Duration of HTN ^1^	N/A	8.6 ± 5.9
SBP, mmHg ^1^	124.0 ± 3.7	155.4 ± 4.8
DBP, mmHg ^1^	82.2 ± 4.1	87.1 ± 2.6
TC, mmol/L ^1^	3.9 ± 0.7	4.8 ± 1.2
LDL-C, mmol/L ^1^	2.5 ± 0.6	3.2 ± 1.1
HDL-C, mmol/L ^1^	1.1 ± 0.3	1.02 ± 0.2
TG, mmol/L ^1^	1.3 ± 0.6	1.5 ± 1.3
CRP, mg/L ^1^	1.07 ± 0.9	7.5 ± 9.6
Brain damage	N/A	(*n* = 2)
Kidney damage	N/A	(*n* = 3)
Eye damage	N/A	(*n* = 2)
CAD	N/A	(*n* = 5)

^1^ Mean ± SD; N/A, not available; SBP, systolic blood pressure; DBP, diastolic blood pressure; TC, total cholesterol; LDL-C, low-density lipoprotein cholesterol; HDL-C, high-density lipoprotein cholesterol; TG, triglyceride; CRP, C-reactive protein; CAD, coronary artery disease.

## Data Availability

The authors confirm that the data supporting the findings of this report are available within the article.
